# Laparoscopic surgery for a hydrocele of the canal of Nuck with an ovarian tumor: An extremely rare clinical finding

**DOI:** 10.1002/ccr3.5320

**Published:** 2022-02-03

**Authors:** Marie Tominaga, Kyoko Morikawa, Yutaro Ogawa, Naomi Kamimura, Ikunosuke Tsuneki, Masaki Tamura, Toru Yanase, Kazuaki Kobayashi, Daisuke Sato, Hitoshi Kameyama, Akira Iwaya, Naoyuki Yokoyama, Shiro Kuwabara, Toshiyuki Yamazaki, Tetsuya Otani, Takumi Kurabayashi

**Affiliations:** ^1^ 26834 Department of Obstetrics & Gynecology Niigata City General Hospital Niigata Japan; ^2^ 26834 Department of Digestive and General Surgery Niigata City General Hospital Niigata Japan

**Keywords:** hydrocele of the canal of Nuck, laparoscopy, ovarian tumor, transabdominal preperitoneal approach

## Abstract

This clinical image presents an unusual report of simultaneous laparoscopic resection of a hydrocele of the canal of Nuck and an ovarian tumor. Laparoscopic treatment with a proper approach is a useful technique in some cases.

## Graphical abstract

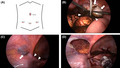

A 21‐year‐old woman had been experiencing a sensation of mass and pain in the left inguinal region. An ovarian tumor was detected by medical checkup echo. Her family and medical histories were unremarkable. The CA 19–9 level was 230.2 U/ml. Other blood data were normal. Magnetic resonance imaging showed a tumor in the right ovary and a tubular cystic lesion in the left inguinal region (Figures 1 and 2).

**FIGURE 1:**
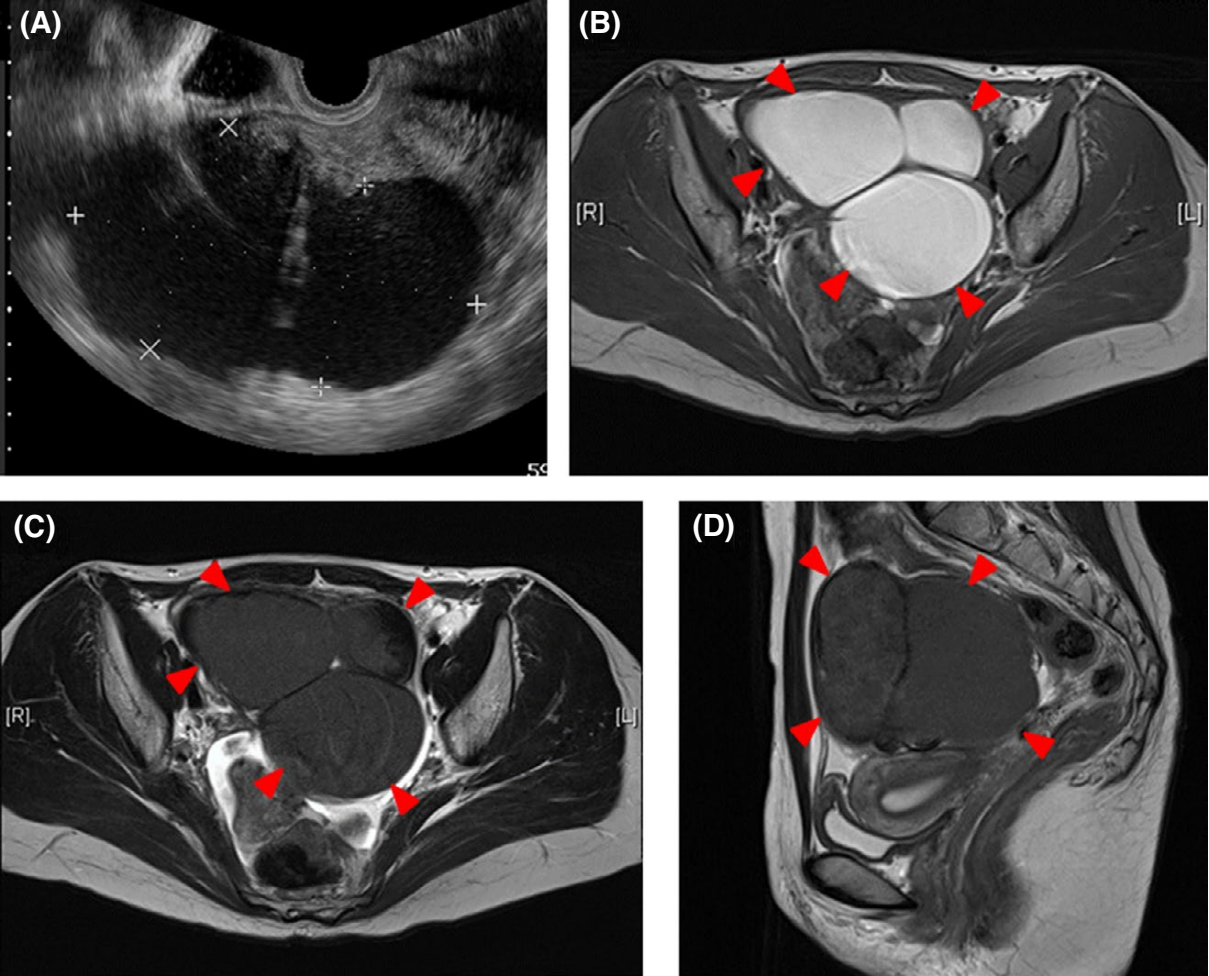
Transvaginal ultrasound and magnetic resonance imaging findings. (A) Transvaginal ultrasound shows cystic pattern with scattered echoes. (B) Transverse T1‐weighted, (C) transverse T2‐weighted, and (D) sagittal T2‐weighted images demonstrate a cystic lesion of 123 × 85 mm in size in the right ovary (red arrowhead)

**FIGURE 2:**
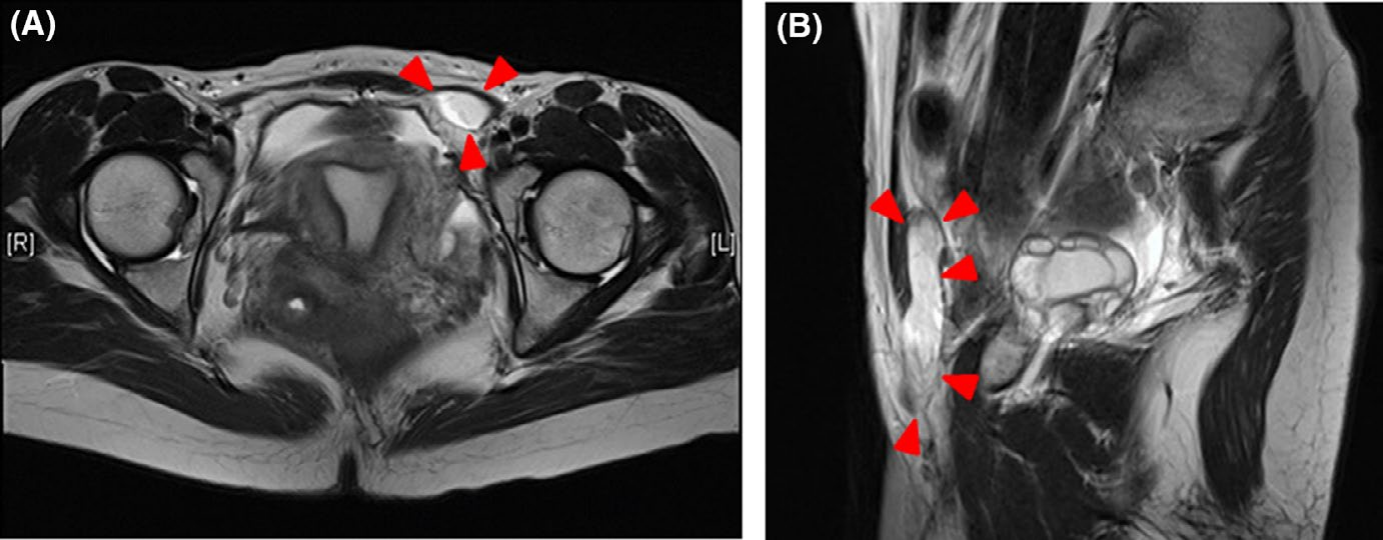
Magnetic resonance imaging findings. (A) Transverse T2‐weighted and (B) sagittal T2‐weighted images demonstrate an elongated tubular cystic lesion of 10 × 50 mm in size found in the left inguinal region (red arrowhead)

She was diagnosed with left hydrocele of the canal of Nuck (HCN) complicated by right ovarian cyst. Since the HCN was located in the inguinal canal and close to the abdominal cavity, simultaneous laparoscopic complete resection was possible using the transabdominal preperitoneal (TAPP) approach (Figure 3). The pathological findings are ovarian endometriotic cyst and HCN (Figure 4). The postoperative course was uneventful, with no recurrence.

**FIGURE 3:**
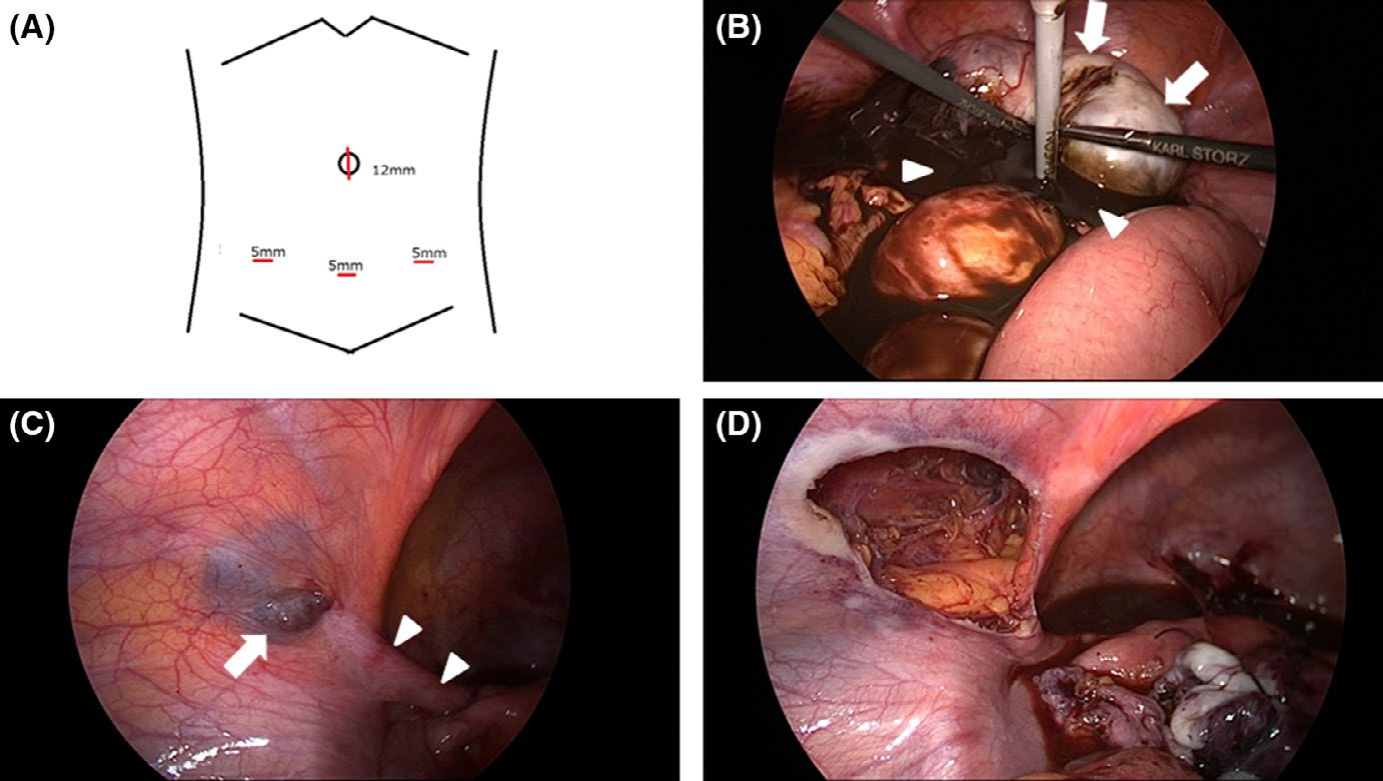
Intraoperative photographs. (A) Procedure was performed in the diamond position. A 12‐mm port was inserted into the umbilicus, and 5‐mm ports were inserted into the midline and right and left sides of the lower abdomen. (B) First, the right ovarian tumor was enucleated (white arrow). During the process, the chocolate‐like contents spilled out (white arrowhead). (C) A dark purple cyst (hydrocele of the canal of Nuck [HCN]) was found in the left inguinal canal, partially protruding into the abdominal cavity (white arrow). Since the HCN was adherent to the round ligament, the round ligament (white arrowhead) was partially resected. (D) Although the HCN was present from the left internal to external inguinal rings, we could dissect and remove the HCN in the inguinal canal from the surrounding area using the transabdominal preperitoneal approach

**FIGURE 4:**
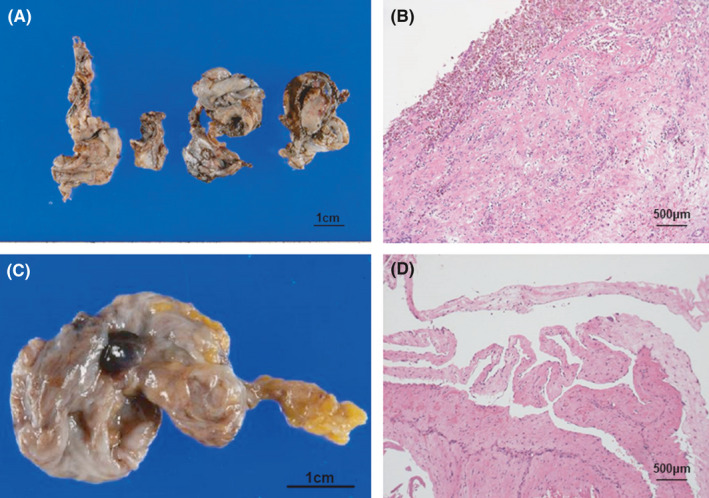
Macroscopic and pathological findings. (A) Macroscopic findings of ovarian cysts. (B) Pathological findings show endometriotic cyst. There were no malignant findings on pathological examination. (C) Macroscopic findings of the hydrocele of the canal of Nuck [HCN]. (D) Pathological findings show an inner wall of cyst comprising mesothelial cells

Hydrocele of the canal of Nuck is a very rare disease in adult women; most cases manifest as a swelling of the groin.^1^ Surgery is mainly reported by inguinal incision, but laparoscopic surgery was also reported recently.^2^ This is the first report of simultaneous laparoscopic resection of HCN and ovarian tumor using the TAPP approach. We believe that laparoscopic treatment is a useful technique because of its curative and minimally invasive nature.

## CONFLICT OF INTEREST

The authors declare that they have no current financial arrangement or affiliation with any organization that may have a direct influence on their work.

## AUTHOR CONTRIBUTIONS

All the authors made substantial contribution to the preparation of this manuscript and approved the final version for submission. MT, KM, and YO drafted the manuscript. MT served as a corresponding author. NK, IT, MT, TY, KK, DS, HK, AI, NY, SK, TY, and TO involved in clinical support. TY involved in careful review of the manuscript.

## CONSENT

Written informed consent was obtained from the patients for the publication of their information and imaging.

## Data Availability

Not applicable.
